# Electric Eels Wield a Functional Venom Analogue

**DOI:** 10.3390/toxins13010048

**Published:** 2021-01-10

**Authors:** Kenneth C. Catania

**Affiliations:** Department of Biological Sciences, Vanderbilt University, VU Station B, P.O. Box 35-1634, Nashville, TN 37235, USA; ken.catania@vanderbilt.edu

**Keywords:** muscle, efferent, venom, electrical, electric organ, prey, predator, evolution, escape

## Abstract

In this paper, I draw an analogy between the use of electricity by electric eels (*Electrophorus electricus*) to paralyze prey muscles and the use of venoms that paralyze prey by disrupting the neuromuscular junction. The eel’s strategy depends on the recently discovered ability of eels to activate prey motor neuron efferents with high-voltage pulses. Usually, eels use high voltage to cause brief, whole-body tetanus, thus preventing escape while swallowing prey whole. However, when eels struggle with large prey, or with prey held precariously, they often curl to bring their tail to the opposite side. This more than doubles the strength of the electric field within shocked prey, ensuring maximal stimulation of motor neuron efferents. Eels then deliver repeated volleys of high-voltage pulses at a rate of approximately 100 Hz. This causes muscle fatigue that attenuates prey movement, thus preventing both escape and defense while the eel manipulates and swallows the helpless animal. Presumably, the evolution of enough electrical power to remotely activate ion channels in prey efferents sets the stage for the selection of eel behaviors that functionally “poison” prey muscles.

My goal in this paper is to highlight the remarkable similarities between one particular form of an eel’s electrical attack [1] and the use of venoms by many predators to cause paralysis by disrupting the neuromuscular junction (e.g., curare or alpha bungarotoxin [2], which block muscle acetylcholine receptors, thus preventing muscle depolarization). Let me start by outlining features of the eel’s strategy that underscore this analogy: (1) this strategy is reserved for use on the most difficult prey; (2) the venom analogue is delivered in conjunction with the eel’s bite; (3) the venom analogue is metabolically expensive and metered by the eel based on prey size and movements (in other words, the amount delivered is proportional to the assessed need, e.g., [3,4,5,6]); (4) the venom analogue is delivered throughout the internal tissues of prey, but has a locus of function at the peripheral nerves and muscles; and most significantly, (5) the venom analogue severely disrupts muscle function, causing subsequent paralysis with a duration proportional to the amount of venom analogue delivered. The venom analogue is, of course, electricity. To appreciate how electricity can be used to this end—and to understand how the eel’s output and behavior could have been selected to produce this result—we must first shift our perspective from the historically vague notion that eels simply “stun” their prey. This in turn requires a brief review of the eel’s electric output and its interaction with the prey’s peripheral nervous system [7,8,9].

Electric eels give off two types of discharges [10,11,12,13,14]: a low voltage probing signal that is insensible to most nearby animals, and a high-voltage discharge that can be used for both offense and defense (it was recently discovered that eels also use their high voltage to probe their environment with active electroreception [15]). Both outputs come as monophasic pulses with a duration (roughly 2 milliseconds) and form similar to an action potential. The two pulses have the same simple waveform, although the high voltage has a much greater magnitude and is delivered at a much higher rate. Whereas the low voltage is given off at a rate of a few pulses per second, the high voltage may be delivered at 400 Hz or more. Given that the high-voltage discharge may be 600 volts for a large eel [16], it is not surprising that electric eels have been of interest to scientists for centuries. Yet, most studies have focused on the eel’s ability to generate electricity, with little attention paid to the effect of the electricity on prey. This likely stemmed from the technical limitations of simultaneously taking measurements from both the eel and prey during encounters.

However, advances in multi-channel data recording and high-speed video systems have allowed for a detailed examination of these predator-prey interactions [7]. One of the most important discoveries was that each of the eel’s high-voltage discharges generates an action potential in the motor neurons of nearby fish [7]. This finding was both an astounding revelation about the mechanism of the eel’s attack, and somewhat obvious in retrospect. It was astounding because it revealed that an electric eel’s motor neurons (through the intermediate of the eel’s amplifying electric organ) activate the muscles in nearby prey. In other words, eels have a form of high-fidelity remote control over the nervous systems of other animals. The finding was somewhat obvious in retrospect, because this mode of motor neuron activation is the same mechanism underlying law enforcement TASERs, commonly used therapeutic transcutaneous electric nerve stimulation [17] (TENS), and even classroom demonstrations using student subjects [18].

Figure 1a,b illustrate the paradigm that was used to investigate the effect of the eel’s high-voltage discharges on prey muscles. A whole (pithed) fish preparation with functional muscles was suspended in an aquarium and attached to a force transducer. The eel was separated from the fish by an electrically permeable agar barrier (this prevented the eel from attempting to eat the preparation during the experiments). High-voltage discharges were easily elicited from the eel by simply feeding it in the adjacent compartment. The addition of a second pithed fish—and a second force transducer—provided both a control and an experimental preparation during the study (note that conditions are identical for the control and experimental preparations in Figure 1c,d).

Figure 1c–e highlight several important features of the eel’s high-voltage attack. First, both fish preparations exhibited nearly identical tension responses, despite the variability in the eel’s interpulse intervals; this is a testament to the efficacy of the eel’s high voltage in activating prey muscles. Second, muscle responses followed patterns that would be predicted from a typical muscle physiology experiment; e.g., high rates of pulses produced a fused tension response, individual pulses produced twitches, and doublets of pulses (arrows) produced disproportionately strong tension responses. Finally, curarizing the fish preparation (Figure 1e, bottom trace) eliminated the tension responses, demonstrating that the eel’s pulses activate the muscles by way of the motor neurons.

With this new view of the eel’s effect on prey, it is helpful to invoke a metaphor. Namely, what is seen on the tension traces by an experimenter is also “seen” by natural selection. With such control over muscle activity, one can easily imagine how an experimenter in a physiology laboratory might devise a strategy to disable the prey’s muscles for a period of time. The same could be said for natural selection acting on eel behavior. The strategy is to deliver multiple trains of high-frequency stimulation to the motor neuron efferent fibers—a procedure commonly used for research [19], and for student laboratories, when studying muscle fatigue. In fact, this is exactly what electric eels do when the need arises [1].

Before describing the details, it helps to put this unique behavior in context by briefly outlining a more typical electric eel attack. Electric eels are gape-limited predators that immobilize nearby animals with a high-frequency volley of high voltage, which causes tetanus [7] through the mechanism described above and illustrated in Figure 1 (left side of panel c). Often, prey can then be immediately swallowed. However, consider also that electric eels live in the Amazon, surrounded by a diverse assemblage of potential prey. Some animals are difficult to subdue and handle, especially if they have a more electrically resistant cuticle [1]. In addition, small electric eels with low power output may have a difficult time handling even the least resistive targets [1].

It is in these difficult cases that eels essentially “poison” prey muscles in a manner analogous to venom. First, the eel grasps any part of the struggling prey tightly in its jaws, and then curls into a circle to bring the negative pole of its huge electric organ to the opposite side of the animal (Figure 2). Thus positioned, theory would suggest that the eel has maximized the effect of its fixed power output on prey, essentially “focusing” the electric field through the target animal. To test this prediction, a specially designed pair of electrodes was inserted into a pithed fish, with the addition of a crossbar that prevented the eel from swallowing the preparation [1]. As shown in Figure 2d, the experiment confirmed the prediction—often, the voltage recorded inside prey more than doubled when the eel curled. Note that any given eel has a fixed high-voltage output that cannot be modulated in amplitude [20,21]. Therefore, the changes in recorded voltage during this experiment are the result of changes to the configuration of the dipole electric field. It might, at first, seem surprising that the voltage more than doubled when the eel curled, but this follows from the fact that the front pole of the electric organ is always at a fixed, minimal distance from the prey due to the intervening head and viscera. In contrast, the caudal pole of the electric organ in the tail can be brought into nearly direct contact with prey to have a stronger effect (the effect of the front and back poles of the electric organ are additive). In any event, the curling behavior allows the eel to maximally stimulate the prey’s internal tissues, ensuring activation of motor neuron efferents.

In addition to confirming the predicted electrical effect of curling, these experiments also serendipitously revealed how eels “meter” their metabolically expensive high voltage. The trigger for the initiation of curling behavior, and the cue for determining the duration of the behavior, is prey movement. For example, jiggling the wires to cause the electrodes to simulate prey movement elicited curling and the accompanying high-voltage volleys. In the absence of movement, the eel would uncurl and attempt to swallow the prey, whereas additional movements would elicit additional curls and high-voltage volleys (an eel might curl in opposite directions in sequential curls). Note that this finding is very similar to the description provided by Wigger and colleagues [3] that spiders detect the vibration caused by insect wings, which “obviously give a signal to the spider that there is a struggling item which might escape”, eliciting the injection of more venom. An identical argument (detection of struggling prey) was previously made for the prolongation of electric eel curling behavior accompanied by high-voltage volleys [1].

The key aspect of this curling behavior, and the focus of this paper, is the details of the high-voltage delivery and its subsequent effect on prey. Once curled, an electric eel gives off repeated volleys of high-voltage discharges at a rate of roughly 100 Hz. This kind of stimulation regimen is commonly used in the laboratory in order to study fatigue [22]. In the laboratory, however, the focus is often on isolated muscle fibers, which appear to fatigue more slowly than whole muscles [19]. In striking contrast to laboratory conditions, electric eels in the amplified, curled position are likely to be stimulating nearly every motor neuron efferent, and therefore nearly every muscle, in the entire animal. This has the potential to produce profound, whole-body fatigue with the largest and fastest contracting muscle fibers failing first [23,24].

To test this possibility, an experiment was arranged that simulated the electric eel’s output when curled [1]. Figure 3 summarizes the procedure and the results. To accurately stimulate a muscle preparation in the same manner as an electric eel, the recording procedure described above was used to elicit curling and to record the eel’s high-voltage discharges. These pulses, in turn, triggered a stimulator that activated the muscle preparation, thus mimicking the rate and timing of the eel’s stimulation pattern. For both whole fish preparations and crayfish tail preparations, the result was rapid loss of contractile force. Presumably, a similar loss of contractile function under natural circumstances would prevent the fast escape that is typical of both of these prey. The analogy to a neuromuscular blockade is brought home when one compares the experiments using curare (Figure 1e) to the results of the eel’s induction of involuntary fatigue (Figure 3e,f)—though it is striking that the eel’s electrical strategy took effect faster [1].

Given that an electric eel already has a grip on prey when this paralyzing technique is used, one might wonder why it is necessary in the first place. The answer becomes obvious when natural predatory encounters are observed, and the limitations on the eel’s prey handling ability are considered. Two types of encounters serve to highlight the utility of the behavior: (1) small electric eels feeding on fish, and (2) larger electric eels feeding on more difficult prey.

Fish are often initially captured lengthwise, or held precariously (by the tail, for example), making them difficult or impossible to swallow without repositioning. Although eels are able to firmly grasp prey, unlike a snake, they cannot manipulate prey without releasing their grip entirely. Fish often escape in the process [1]. The solution for small eels is to curl around the fish and cause short-term immobility by delivering a series of amplified volleys immediately prior to releasing the fish for subsequent swallowing. Figure 3g–j illustrates a successful capture using this strategy. Notice that the fish is entirely released (red arrow, Figure 3i) in the process and could have easily escaped if able to move. Short-term paralysis is sufficient for swallowing in this situation [1].

Large electric eels also use this technique when presented with particularly difficult prey. For example, when large crayfish with large claws were offered to a large electric eel, the eel invariably used the curling technique and extended the duration of the curled configuration for nearly a minute, all the while delivering repeated volleys of high-voltage pulses as described above (not illustrated, but see [1]). Because of the asymmetric nature of crayfish muscles, the effect on claws and limbs was obvious, i.e., the claws and limbs contracted with each volley, until over time the limbs became flaccid and immobile (see supplementary movies in [1]). It is all but certain that electric eels are able to monitor muscle responses with the sensitive tissues of their mouth while holding the prey for curling, and this likely elicited the long volleys observed for crayfish. In fact, shortly after the crayfish limbs became flaccid, the eel repositioned the crayfish multiple times and then swallowed it whole. In the case of such potentially dangerous prey, the induction of longer term, involuntary fatigue may not only prevent escape, but also prevent injury from an animal attempting to defend itself. Crayfish that are removed from the eel before being eaten recover their mobility over time. On some occasions, eels catch two fish before curling, and manage to swallow only one. On those occasions, the remaining fish also recovered [1].

It is possible that, in addition to inducing involuntary fatigue, large electric eels with the most powerful electric organs cause additional disruptions to the nervous system and muscles, which help to incapacitate prey. However, this does not detract from the venom analogy, as many venoms are aptly described as a “witch’s brew” of components (e.g., alpha and beta neurotoxins) that work together to efficiently immobilize prey—or on other occasions, to deter potential predators. There is no doubt, for example, that the purpose of a different electric eel behavior—the leaping defensive shock [25,26]—functions by causing brief, but severe, pain as a deterrent. In this sense, electric eels use their high voltage to cover the “main axes” of venom function suggested by Neirmann et al. (in this Special Issue [27]), “immobility vs. pain”. Both are achieved by the eel, but under very different circumstances. Moreover, electric eels provide a key example of deterrence through pain without causing harm (the author can attest to the efficiency of the former in the absence of the latter [28]), perhaps informing hypotheses regarding the range of venom functions that might be expected to evolve [27].

In closing, I do not suggest that the eel’s electrical attack falls under the technical umbrella of venom. Rather my goal is to highlight the remarkable similarity in functions that have evolved from a similar underlying substrate—the ability for one animal to remotely activate the ion channels of another. Nature is replete with organisms that achieve this result through chemical means, and it is remarkable that the same end can be achieved with electricity.

## Figures and Tables

**Figure 1:**
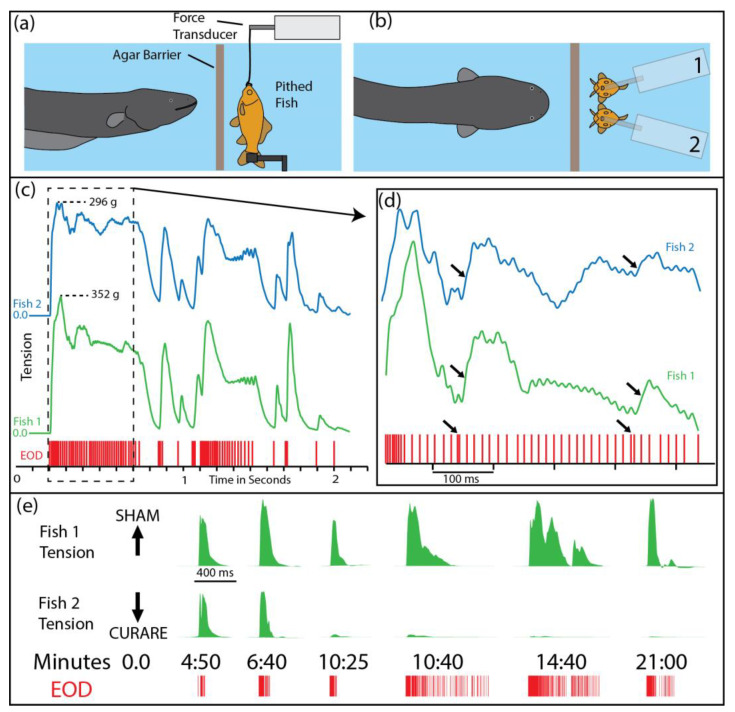
Paradigm and results used to determine the effect of electric eel high-voltage discharges (EOD) on prey muscles. (**a**) A pithed fish with working muscles was attached to a force transducer and separated from the eel by an electrically permeable agar barrier. (**b**) Elaboration of the paradigm by adding an additional fish and force transducer provided a control and experimental preparation (the two preparations are treated identically in “(**c**–**d**)”, see “(**e**)” for curare treatment of one preparation). (**c**) Tension traces from the 2 fish preparations (blue and green) in response to high-voltage impulses (red tick marks below, labeled EOD) generated by the eel while being fed in the adjacent chamber. Note the remarkable similarity of tension responses. The whole-body tetanus indicated on the left side is typically equivalent to the maximal tension that can be obtained by direct connection to an external stimulator (see [7]). (**d**) An expansion of the data trace in “(**c**)” showing the effect of doubles (arrows). (**e**) Reponses over time showing the effect of curare (lower trace) compared to a sham injection (upper trace). Curare acts by blocking the acetylcholine receptors and hence preventing synaptic communication between the motor neuron and the muscle. Tension responses were eliminated by curare, indicating that electric eels activate muscles by way of the motor neurons. EOD = electric organ discharge. Panels in the figure modified from [7] with permission.

**Figure 2:**
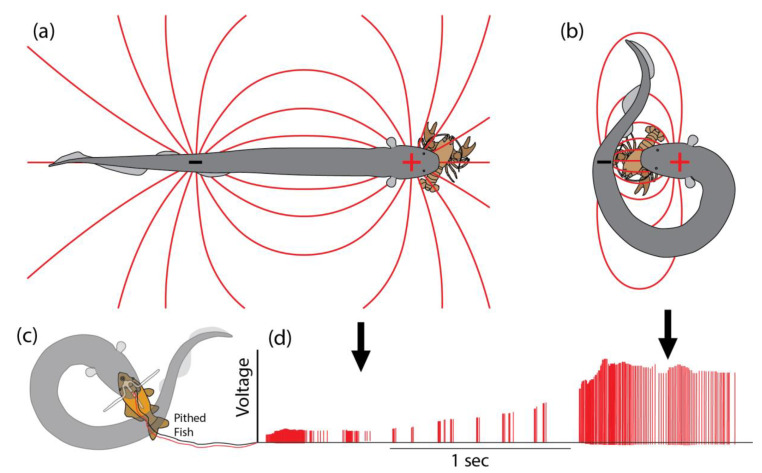
How electric eels amplify the effect of their fixed power output. (**a**) The dipole field that surrounds an electric eel with its body extended. (**b**) When the eel brings the negative pole (the tail) around and behind the prey, the dipole field is reconfigured to have the strongest effect on prey held tightly in the eel’s mouth. This configuration ensures maximal stimulation of the prey efferents and may be most important when prey have a resistive cuticle. (**c**) Apparatus for measuring the change in electrical potential within prey as the eel changes position to reconfigure the dipole field. The pithed fish is impaled on a plastic spike containing electrodes, while a crossbar prevents swallowing. Movement of the wires to simulate prey movement elicits the curling behavior and high-voltage volleys. (**d**) Example of the change in voltage within prey as the eel changes from the linear position (left arrow) to the curled position (right arrow). Note that any given eel has a fixed high-voltage power output, and therefore the increase in voltage reflects changes in the configuration of the electric field (see text). Panels above modified from [1] with permission.

**Figure 3:**
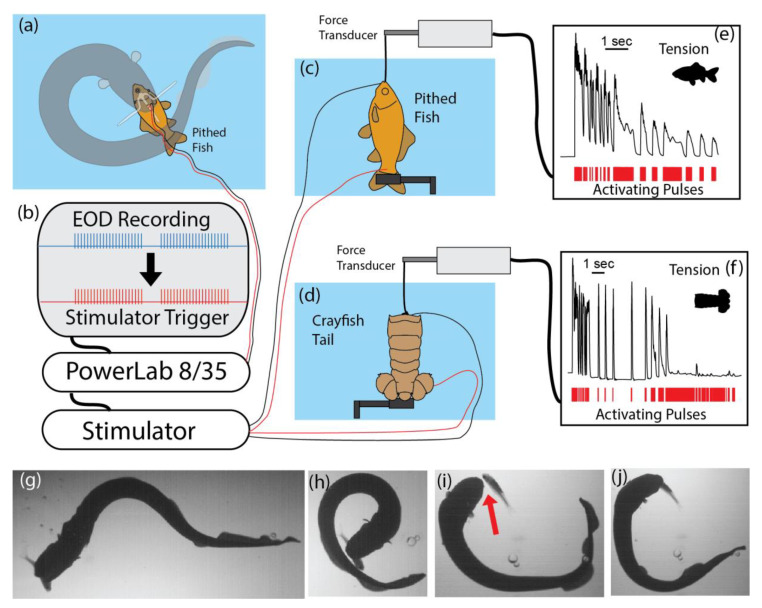
Paradigm for measuring the effect of multiple high-voltage volleys on prey muscles. (**a**) The electrodes used to record an electric eel’s output while curled. (**b**) Schematic of the equipment used to activate a stimulator that precisely mimicked the rate and pattern of eel discharges in real-time. (**c**) Fish preparation connected to force transducer. (**d**) Crayfish tail preparation connected to force transducer. (**e**) Change in fish tension response over the course of stimulation. (**f**) Change in crayfish tail tension response over the course of stimulation. (**g**–**j**) Frames captured from video showing a small electric eel using the curling strategy to immobilize a fish prior to releasing its hold (red arrow in “(**i**)”) so that it can swallow head first. See [1] for more details. Some panels modified from [1] with permission.
